# Editorial: Language, Cognition, and the Manipulated Brain: Theoretical and Experimental Perspectives on Manipulative Processes in Language Comprehension

**DOI:** 10.3389/fpsyg.2022.869595

**Published:** 2022-03-10

**Authors:** Viviana Masia, Davide Garassino, Louis de Saussure, Nicola Brocca

**Affiliations:** ^1^Department of Languages, Cultures, Literature and Translation, Sapienza University of Rome, Rome, Italy; ^2^Institute of Romance Languages, University of Zurich, Zurich, Switzerland; ^3^Department of Applied Linguistics, Institute of Translation and Interpreting, Zurich University of Applied Sciences (ZHAW), Zurich, Switzerland; ^4^Institut des Sciences du Langage et de la Communication, Université de Neuchâtel, Neuchâtel, Switzerland; ^5^Department on Subject-Specific Education, University of Innsbruck, Innsbruck, Austria

**Keywords:** manipulation, cognition, pragmatics, brain, implicit communication

Manipulation is among the most recurrent topics in argumentation studies (Masia; de Saussure, 2005; Maillat, 2013; Oswald et al., 2016; Sorlin, 2017 among others). Most of what we know today about deceptive and manipulative uses of language seems to involve the impact that vagueness, ambiguity, presupposition, implicature and other types of underencoded meanings wield on sentence comprehension as well as of people's likelihood of complying with the manipulator's intentions. It is generally concurred with that the success of manipulation lies on the addressee's failure to detect it (de Saussure, 2005). This is quite often the case of several types of manipulative discourse (e.g., advertising and political speech, among others), where the speaker's deceptive communicative aims are pursued by means of implicit discourse strategies. Indeed, it seems that through linguistic implicitness, the speaker can retain relevant contents and release less relevant ones. This makes it extremely difficult, if not impossible, for the addressee to cautiously verify the truth of some negotiated content, thereby challenging it, if necessary.

The cognitive underpinnings of manipulation have also been the plank of current empirical research, involving processing-based tests up to neurophysiological inquiries which seek to unravel both the mental operations entailed in the recognition of manipulative linguistic strategies and the type of brain activity elicited when manipulative communication has to be detected. All in all, these lines of experimental research explore the scope and boundaries of the well-known *mind-reading* module, that is, the cognitive equipment that allows a human being to access her interlocutor's mental states as well as pin down those hidden communicative intentions which are conveyed through other literally expressed content.

With a view to shedding light on these and other research paths related to the way the human mind deals with manipulative language, this collection gathers contributions on linguistic phenomena and experimentations variously correlated with manipulative communication. Some inquire about the role of metaphorical expressions (Dong and Duan's review of Zoltán Kövecses' monograph), others consider the function of presuppositions and different patterns of information structure in the mental encoding of implicit meaning (Lombardi Vallauri), others the use of (un-)certainty expressions depending on one's actual state of knowledge (Lorson et al.). Some of these contributions are more strongly theory-driven (Reboul) and seek to draw fruitful conclusions on the relation that manipulative language bears to human cognition. Other articles present behavioral (Müller and Mari; Yang et al.) and/or neuropsychological experimental findings (Bian et al.) which seek to better gauge the association of specific processing patterns to dealing with certain phenomena of implicit or underencoded communication.

Dong and Duan propose a review of Zoltán Kövecses' new monograph *Extended Conceptual Metaphor Theory* which revisits Lakoff's traditional Conceptual Metaphor Theory. A crucial issue broached in the volume concerns the role of context-sensitivity parameters in metaphor comprehension, which also provides a fruitful groundwork to further speculate on the effects of metaphor processing in manipulative discourse.

Zooming in on how persuasive strategies are used to convey questionable contents, Lombardi Vallauri addresses the function/s of presupposition and topic of inducing shallower processing of some information, as also backed up by earlier and recent behavioral studies. From a neurophysiological perspective, though, Lombardi Vallauri's contribution also accounts for deflecting processing scenarios, whereby presupposition and topic would elicit costlier cognitive operations, when associated to new contents in an utterance. In the domain of EEG research, such increasing costs are manifested in more prominent deflections in the N400 and/or P600 components as well as in synchronous or asynchronous oscillations in different frequency bands. According to the author, the effort devoted to accommodating new implicit contents may drain resources from critical evaluation, resulting in shallower processing.

Bian et al. describe the results of an ERP study on attraction effects in advanced second language learners of English. The authors report stronger P600 effects in response to ungrammatical verb agreement, which were replaced by N400 when a NP attractor interrupted the subject-verb relation. In their account, while N400 effects have been interpreted as hinting at shallow and more heuristic processes stemming from the evaluation of lexical associations between agreeing elements, P600 has been seen as indexing a full, combinatorial process responsible for parsing morphosyntactic features between agreement controllers and targets. The research hypotheses of this study provide valuable insights into the functional significance of electrophysiological components with respect to linguistic manipulation of sentence information.

Another intriguing investigation on the interplay between second language acquisition and manipulative communication is the experiment conducted by Yang et al. on Chinese students asked to use dishonest communication in their native language and in English in different tasks. Interestingly, recourse to lying and dishonest communication was much more frequent in the native language condition than in the foreign language condition. Yang et al. correlated this pattern to the fact that lesser proficiency in a language may discourage speakers to use deceptive discourse strategies because they would impose more taxing mental operations to be carried out.

Müller and Mari's contribution reports the results of a self-paced reading task and of an eye-tracking experiment aimed at assessing the processing of informative definite descriptions in plausible vs. implausible contexts. As a replication of Singh et al.'s (2016) study in French, the authors wanted to demonstrate that definite descriptions are significantly costlier when they occur in implausible contexts. Since no significant differences emerged in eye-tracking measures between plausible and implausible conditions, the authors suggested that, in online processing, participants first adopted a stance of trust to understand utterances, and only then did they filter the information through their epistemic vigilance module.

The study run by Lorson et al. investigated speakers' motivations in choosing between (un)certainty expressions such as “believe” or factive verbs like “know.” Notably, the authors sought to unravel whether the choice of more or less certain expressions is conditional upon (i) how likely an event is estimated to be and (ii) other strategic aspects of the communicative context in which an interaction takes place. The second experiment precisely focused on the use of the (un)certainty expressions “know” and “believe” using the same testing protocol but having participants only use these two predicates in their reports. What emerged from this study is that not only did participants use (un)certainty expressions depending on their degree of belief, but they also adjusted such use based on the communicative situation at hand.

Reboul proposes an insightful reflection on how underinformativity would enhance the persuasive effects of a message reducing the sender's likelihood of being punished if her message turns out to be untrue. Through underinformativity, speakers can indirectly communicate false contents while producing an utterance that is literally true, what Reboul calls *truthfully misleading*. The effectiveness of this strategy rests upon the fact that part of the responsibility for the false content is deferred to the hearers, and this legitimizes a speaker to appear as having been misunderstood in the communication process.

## Author Contributions

All authors listed have made a substantial, direct, and intellectual contribution to the work and approved it for publication.

## Conflict of Interest

The authors declare that the research was conducted in the absence of any commercial or financial relationships that could be construed as a potential conflict of interest.

## Publisher's Note

All claims expressed in this article are solely those of the authors and do not necessarily represent those of their affiliated organizations, or those of the publisher, the editors and the reviewers. Any product that may be evaluated in this article, or claim that may be made by its manufacturer, is not guaranteed or endorsed by the publisher.
